# Charged Gold Nanoparticles Promote In Vitro Proliferation in *Nardostachys jatamansi* by Differentially Regulating Chlorophyll Content, Hormone Concentration, and Antioxidant Activity

**DOI:** 10.3390/antiox11101962

**Published:** 2022-09-30

**Authors:** Shubham Joshi, Aqib I. Dar, Amitabha Acharya, Rohit Joshi

**Affiliations:** 1 Division of Biotechnology, CSIR-Institute of Himalayan Bioresource Technology (CSIR-IHBT), Palampur 176061, Himachal Pradesh, India; 2 Academy of Scientific and Innovative Research (AcSIR), CSIR-HRDC Campus, Ghaziabad 201002, Uttar Pradesh, India

**Keywords:** gold nanoparticles, micropropagation, transcript analysis, *Nardostachya jatamansi*, antioxidant activity

## Abstract

*Nardostachys jatamansi* is a critically endangered medicinal plant and endemic to the Himalayas, having high commercial demand globally. The accumulation of various secondary metabolites in its shoots and roots with antioxidant potential are well-documented in traditional as well as modern medicine systems. In the present study, we first attempted to investigate the impact of citrate (−ve charge, 11.1 ± 1.9 nm) and CTAB (+ve charge, 19.5 ± 3.2 nm) coated gold nanoparticles (AuNPs) on the in vitro proliferation and antioxidant activities of *N. jatamansi*. Both the nanoparticles differentially affected the morphological and biochemical parameters, chlorophyll content, internal hormone concentration, and antioxidant activities in a concentration-dependent (10–100 µM) manner. Vigorous shooting was observed in half strength MS medium supplemented with IAA (1 mg/L) with 60 µM citrate-AuNPs (46.4 ± 3.7 mm) and 40 µM CTAB-AuNPs (42.2 ± 3.2 mm). Similarly, the maximum number of roots (5.00 ± 0.67 and 5.33 ± 0.58) and root length (29.9 ± 1.5 mm and 27.3 ± 4.8 mm) was reported in half-strength MS medium with IAA (1 mg/L) supplemented with 60 µM citrate-AuNPs and 40 µM CTAB-AuNPs, respectively. In addition, plants growing on MS medium supplemented with 60 µM citrate-AuNPs and 40 µM CTAB-AuNPs showed significantly enhanced photosynthetic pigments (chlorophyll a and b, carotenoids, and total chlorophyll), internal hormone concentration (GA3, IAA, and ABA), and antioxidant activities (total phenolics, flavonoids, DPPH, and SOD enzyme activity). Moreover, the transcript analysis of *ANR1*, *ARF18*, *PLY9*, *SAUR28*, *GID1A*, *GRF1*, *SOD*, and *CAT* further confirmed the role of 60 µM citrate-AuNPs and 40 µM CTAB-AuNPs in the improvement in the growth and antioxidant activities of *N. jatamansi.* Bearing in mind the urgent requirements of the effective conservation measures of this endangered species, the present findings suggest the elicitation of citrate-AuNPs and CTAB-AuNPs would significantly improve the potential applications of *N. jatamansi* in the medicinal plant-based industry.

## 1. Introduction

*Nardostachys jatamansi* is a critically endangered, perennial medicinal herb of the family Caprifoliaceae (https://www.iucnredlist.org/species/50126627/50131395, accessed on 16 July 2014), and is restricted to the Alpine Himalayas at an altitude of 3000 m to 5000 m above sea level [1]. As an adaptation to the high altitude cold region, this species primarily propagates through its underground rhizomes and has a 3–4 year juvenile phase, followed by a short reproductive stage and poor seed germination rate [2]. Since it is not cultivated, the complete demand of raw material is fulfilled through the indiscriminate and extensive illegal trading of plant material, leading this species to the verge of extinction. The majority of its secondary metabolites have been reported in the rhizomes and roots, although few have also been reported in the leaves [3]. These secondary metabolites are used as curative agents and natural ingredients by the cosmetic, nutraceutical, and pharmaceutical industries. The immense commercial value of this species is because of its sedative, neuroprotective, hepatoprotective, antidiabetic, cardioprotective, anti-lipid peroxidative, hypolipidemic, antioxidant, tranquilizing, hypotensive, anti-inflammatory, antidepressant, anticonvulsant, antiasthmatic, antiestrogenic, and anticancer properties [4,5]. Thus, to improve its propagation for the conservation of this valuable Himalayan plant and to reveal its medicinal potential, researchers are exploring alternative approaches.

To survive in an alpine region, *N. jatamansi* is well-adapted for adverse climatic conditions with improved protective mechanisms (i.e., efficient photosynthesis, photoprotection, and antioxidant levels) that help him to proliferate in their natural habitats [2]. In addition, the overproduction of the reactive oxygen species (ROS) is a direct consequence of cold stress induced cellular response, which leads to hyperosmotic stress and ionic imbalance through oxidative damage to the membrane lipids, pigments, proteins, and DNA, causing cell death [6]. To prevent the toxicity effect of ROS, cells have evolved an array of enzymatic (SOD, CAT, APX, GR, and POX) and non-enzymatic antioxidants (e.g., α-tocopherol, ascorbate, glutathione) [7]. However, limited information is available on the production and mitigation of ROS in high-altitude medicinal plants. Nanotechnology is an emerging area, which can be used to explore the cellular responses and antioxidant regulatory mechanisms in high-altitude medicinal plants.

Nanotechnology has the potential to maintain sustainable agriculture practices, and metal-based nanoparticles (NPs) have shown promising results for crop growth [8]. Gold nanoparticles (AuNPs) have an inert core material; thereby, these can be functionalized with surface coatings to prevent the interaction between gold and its surrounding environment [9]. It was demonstrated earlier that low concentrations of AuNPs enhance the antioxidant enzyme activity, photosynthetic pigment content, electron transport rate, water metabolism, and the growth of plants [10]. Although gold nanoparticles can accumulate in the plants and have the potential to alter plant growth and secondary metabolism, it is necessary to understand their interaction with plants [11]. At present, numerous protocols for obtaining artificial AuNPs with different properties, shapes, and sizes have been developed, and the accessibility of these methods makes the wider utility of AuNPs in different agricultural practices. Thus, the use of unique nanoparticles with defined physical and chemical properties has arisen [12]. However, surface modifications could stabilize nanomaterials, though under natural conditions, the chemistry of coating can change, affecting the properties and biocompatibility of the nanoparticles [13].

Therefore, considering the above facts, the present investigation was aimed to investigate whether the charge on the surface of the plant cell wall or non-specific binding, along with particle size, improves the in vitro regeneration potential and antioxidant activity of *N. jatamansi*. To the best of our knowledge, there is no prior study available on the impact of nanoparticles on Himalayan medicinal plant *N. jatamansi*. This technique will offer unexplored possibilities to improve the growth in high altitude medicinal plants. The present study was designed to (1) determine the optimum concentration of citrate coated gold nanoparticles (citrate-AuNPs) and CTAB coated gold nanoparticles (CTAB-AuNPs) to promote the shoot and root proliferation under in vitro conditions; (2) study the changes in photosynthetic pigment contents, antioxidant enzyme activities, accumulation of phenolic compounds and internal hormone concentration in citrate-AuNPs and CTAB-AuNPs treated plants; and (3) evaluate the differential accumulation of various transcripts related to the growth and antioxidant activities.

## 2. Materials and Methods

### 2.1. Materials and Reagents

Chloroauric acid (HAuCl_4_.3H_2_O), cetyltrimethylammonium bromide (CTAB), and ascorbic acid were purchased from Merck Sigma-Aldrich, (Bangalore, India). Trisodium citrate and sodium hydroxide (NaOH) was procured from SDFCL, (Mumbai, India). Thidiazuron (TDZ), kinetin (Kn), indole-3-Acetic acid (IAA), sucrose, and agar was procured from HiMedia, India. HPLC grade IAA, GA3, and ABA was procured from Merck Millipore, (Mumbai, India) with a ≥95% purity.

### 2.2. Synthesis of Citrate-AuNPs

Citrate-AuNPs were synthesized according to [14], with slight modifications. For this, 10 mL of 5 mM gold (III) chloride trihydrate salt (HAuCl_4_·3H_2_O) was diluted in 190 mL Mili-Q water. The resulting solution was then heated at 90 °C under inert and stirring (700 rpm) conditions on a magnetic stirrer. To this boiling solution, ~4 mL of 20 mM tri-sodium citrate dihydrate (Na_3_C_6_H_5_O_7_·2H_2_O) was added drop-wise using a dropping funnel. The color of the solution turned from colorless to wine red, indicating the formation of citrate-AuNPs. Finally, the wine red colored solution was dialyzed overnight to remove the unreacted reactants and stored at room temperature.

### 2.3. Synthesis of CTAB-AuNPs

The CTAB-AuNPs were synthesized using a one-step reaction according to the previous literature [15]. Briefly, 100 μL of 50 mM solution of gold (III) chloride trihydrate salt (HAuCl_4_·3H_2_O) was prepared in Mili-Q water and added to 40 mL of CTAB (200 mM) in a dropwise manner. After this, 120 μL of pre-cooled ascorbic acid (~75 mM) was carefully added to this solution until the color of the solution turned from yellowish to colorless. Furthermore, 500 μL of 100 mM sodium hydroxide (NaOH) solution was added to this solution. Finally, the color of the solution started changing to wine red, which indicated the formation of CTAB-AuNPs. To remove the unreacted moieties, these NPs were then centrifuged thrice at 12,000 rpm for 20 min and redispersed in Mili-Q water for further characterization and stored at room temperature.

### 2.4. Characterization of Synthesized AuNPs

Absorption studies of citrate-AuNPs and CTAB-AuNPs were carried out using a NanoDrop 2000 Spectrophotometer (Thermo Fisher Scientific, Waltham, MA, USA) in a 1 cm quartz cell and the total volume in each titration was made to 3 mL. The hydrodynamic diameter and net zeta potential of the prepared AuNPs was determined by using a DLS/Zeta sizer (Zetasizer Nano ZS, Malvern Instruments Ltd., Malvern, UK). All of the measurements were recorded by taking 1 mL of the NP solution. For analysis, readings were taken in three technical replicates per sample. For TEM analysis, ~20 µL of the AuNP solution was directly drop-casted on the carbon coated copper grid and kept for drying overnight. The images were captured using Tecnai T20 twin, TEM 200 kV, FEI Netherlands.

### 2.5. Plant Material and Treatment Conditions

Shoot cultures of *N. jatamansi* were obtained from the Division of Biotechnology, CSIR-Institute of Himalayan Bioresource Technology, Palampur, India from the previous researchers and maintained in a previously standardized MS medium [16] with 0.75 mg/L TDZ and 0.5 mg/L kinetin under in vitro condition [5]. Established shoots of 0.5–1 cm height were sub-cultured on previously defined rooting medium containing half strength MS medium, 30 g/L sucrose, 8 g/L agar, and the pH was maintained at 5.75 ± 0.2. After autoclaving at 121 °C and 15 psi for 20 min, media were supplemented with 1 mg/L IAA and different concentrations (10–100 µM) of citrate-AuNPs and CTAB-AuNPs under an aseptic environment. Each treatment consisted of a biological triplicate with three explants per flask. The flasks were incubated at 25 °C ± 2, with a 16/8 h illumination period for 30 days.

### 2.6. Morphological Analysis

In vitro grown plants of *N. jatamansi* were harvested from the medium after 30 days. Photographs were taken and maximum root and shoot length was measured using ImageJ software. The number of roots per explant and the total plant biomass (fresh weight) were also measured manually.

### 2.7. Chlorophyll Estimation

Total chlorophyll, chlorophyll a and b, and total carotenoid content were measured using a method defined earlier [17]. Briefly, 0.1 g tissue was extracted in 80% acetone (Merck Millipore), centrifuged at 10,000 rpm for 10 min (LEGEND MICRO 21R centrifuge, Thermo Fisher Scientific, Waltham, MA, USA). The supernatant was transferred to a fresh tube, and extraction was repeated until the solution became colorless. The absorbance was measured at 663, 645, and 480 nm using UV–Visible spectrophotometer against a blank and photosynthetic pigments were calculated as described earlier [18].

### 2.8. Total Soluble Sugar Content

The anthrone method was used to analyze the total soluble sugar [19]. Briefly, 0.2 g of tissue was extracted with 1 mL of deionized water and boiled for 20 min, and centrifuged at 12,000 rpm for 10 min (LEGEND MICRO 21R centrifuge, Thermoscientific, Waltham, MA, USA). Furthermore, 1.8 mL of deionized water with 0.14% anthrone (Merck Sigma-Aldrich, Bangalore, India) in 2 mL H_2_SO_4_ (Merck Millipore, Mumbai, India) was mixed with 0.2 mL supernatant, boiled for 20 min, and the absorbance was measured at 620 nm. The glucose standard curve at 620 nm with the linear equation Y = 1.255X + 0.1133 was used to determine the total soluble sugar content in each sample.

### 2.9. Estimation of Non-Enzymatic Antioxidants

The methanolic extract was prepared using the control and treated leaves. A total of 100 mg of fresh leaf samples were mixed with 10 mL 80% methanol (Merck Millipore, Mumbai, India), and rotated overnight at 150 rpm. The resultant extract was centrifuged and filter sterilized for the further assessment of phenolics, flavonoids, and % DPPH inhibition. For the estimation of total phenolic content, 1 mL of extract was combined with 2.5 mL of 10% Folin–Ciocalteu (HiMedia, Mumbai, India, 25%) and 2 mL of 2% sodium carbonate (HiMedia, Mumbai, India), incubated for 30 min in the dark, and the absorbance was measured at 765 nm. The standard curve of gallic acid (Merck Sigma-Aldrich, Bangalore, India ≥98.0%) with linear equation Y = 4.8744X − 0.0235 was used to express the results as the total phenolic content mg/g of the total extract [20]. The estimated total flavonoid content, the extract of 0.15 mL was mixed with 5% NaNO_2_ (HiMedia, Mumbai, India), and 0.6 mL of distilled water, incubated at room temperature for 5 min. Furthermore, 0.045 mL of 10% AlCl_3_ (HiMedia, Mumbai, India) was added to the mixture and incubated for 1 min, 0.3 mL of 1 N NaOH (HiMedia, Mumbai, India), and 0.3 mL of deionized water was further added after incubation, and the absorbance was measured at 510 nm. The standard curve of quercetin (Merck Sigma-Aldrich, Bangalore, India, ≥95%) with the linear equation Y = 2.954X + 0.0577 was used to express the results as the total flavonoids mg/g of the total extract [20].

### 2.10. DPPH Radical Scavenging Activity

An extract of 0.5 mL was added to 3.5 mL of 1, 1-diphenyl-1-picrlhydrazyl (DPPH) (Merck Millipore, Mumbai, India, ≥90%), incubated for 30 min in the dark, and the absorbance was recorded at 517 nm. Percent inhibition was calculated through decreasing absorbance by the following equation:

Inhibition % = (Abs (blank) − Abs (sample))/(Abs (blank)) × 100

As the morphological growth parameters and non-enzymatic antioxidant activity were significantly affected in the 10 µM and 60 µM concentrations of the citrate-AuNPs treated plants, and 40 µM concentration of the CTAB-AuNP treated plants, further endogenous hormones and transcript regulation were analyzed in the above said concentrations along with the control.

### 2.11. Superoxide Dismutase (SOD) Enzyme Activity

For the determination of SOD enzyme activity, the control and treated leaf samples were homogenized in liquid nitrogen. Potassium phosphate buffer of 50 mM (pH 7.5) and EDTA (Sigma-Aldrich) 0.5 mM was further added to the resultant mixture, centrifuged at 12,000 rpm (LEGEND MICRO 21R centrifuge, Thermo Fisher Scientific, Waltham, MA, USA) for 15 min, and the supernatant was stored at 4 °C. For activity analysis, 0.1 mL of extract was mixed with 75 µM NBT (Merck Sigma-Aldrich, Bangalore, India), 14.5 mM methionine (Merck Sigma-Aldrich, Bangalore, India), 50 mM sodium carbonate (Merck Sigma-Aldrich, Bangalore, India), 0.1 mM EDTA, 50 mM potassium phosphate buffer, and 4 µM riboflavin (Merck Sigma-Aldrich, Bangalore, India), incubated under fluorescent light for 15 min. Extract free enzyme tube was considered as the control and the non-incubated mixture was used as the blank. After incubation, the absorbance was recorded at 560 nm and one unit of enzyme activity was defined as the reduction of absorbance by 50% in comparison to the control [21].

### 2.12. Endogenous Hormone Content Measurement

The quantitative analysis of IAA (indole-3-acetic acid), GA3 (gibberellic acid), and ABA (abscisic acid) was performed by the method as described earlier [22]. Briefly, 0.1 g leaf samples from the GNPs treated as well as the control plants were homogenized in liquid nitrogen and transferred to a 2 mL centrifuge tube. Each tube was filled with 0.5 mL of extraction solvent, consisting of 2-propanol/distilled water/concentrated HCl (2:1:0.002). Tubes were rotated at a speed of 100 rpm at 4 °C for 30 min. One mL of dichloromethane (Merck Millipore, Mumbai, India) was then added to the mixture and further rotated for 30 min at 4 °C. The resultant mixture was centrifuged at 11,000 rpm for 5 min, and 0.9 mL of the lower phase was transferred into a centrifuge tube. The solvent mixture was then dried in a lyophilizer and the resultant samples were dissolved in 0.1 mL of methanol. A total of 50 µL of the samples was injected into the C18 reverse phase column for UPLC analysis.

### 2.13. RNA Isolation and qRT-PCR Analysis

RNA was isolated in triplicate by the iRIS method [23] from the leaves of the citrate-AuNP and CTAB-AuNP treated plants along with the control. Isolated RNA was quantified using a NanoDrop Spectrophotometer. First-strand cDNAs were then synthesized using the Verso cDNA Synthesis Kit (Thermo Fisher Scientific, MA, USA) as per the manufacturer’s instruction. For expression analysis, the contigs of *PYL9*, *ARF18*, *ANR1*, *SAUR28*, *GRF1*, and *GID1A* were extracted from the online transcriptomic database of *N. jatamansi* (SRR1241890) and contigs of *CAT1* and *SOD1* were extracted from the SRR11184224 database [24] (Supplementary Figure S1). Further using these contig sequences, primers were designed through the primer 3 online software (Table 1). Finally, qRT-PCR in three biological replicates of each sample was completed using the ABI StepOne Real-Time PCR instrument by Applied Biosystems. A 10 µL of the reaction mix was prepared using the DyNAmo color flash SYBR green master mix Thermo Fisher Scientific, MA, USA, as per the manufacturer’s instructions. The amplification cycle comprised of 95 °C for 7 min initiation, followed by an amplification step of 40 cycles at 95 °C for 10 s, and 60 °C for 30 s; the melting curve was analyzed with default reaction cycles. The relative quantification method (2−ddCT) was used to quantify the variations using actin as an internal control according to the previously published literature [24].

### 2.14. Statistical Analyses

All statistical analyses were carried out, and bar graphs were assembled using SIGMA PLOT v 14.5 scientific software. In all experiments, the significance levels were determined using the two-tailed Welch’s *t*-test of *p* < 0.05 and examined between the control and treated plants [25,26].

## 3. Results

### 3.1. Characterization of AuNPs

**Figure 1:**
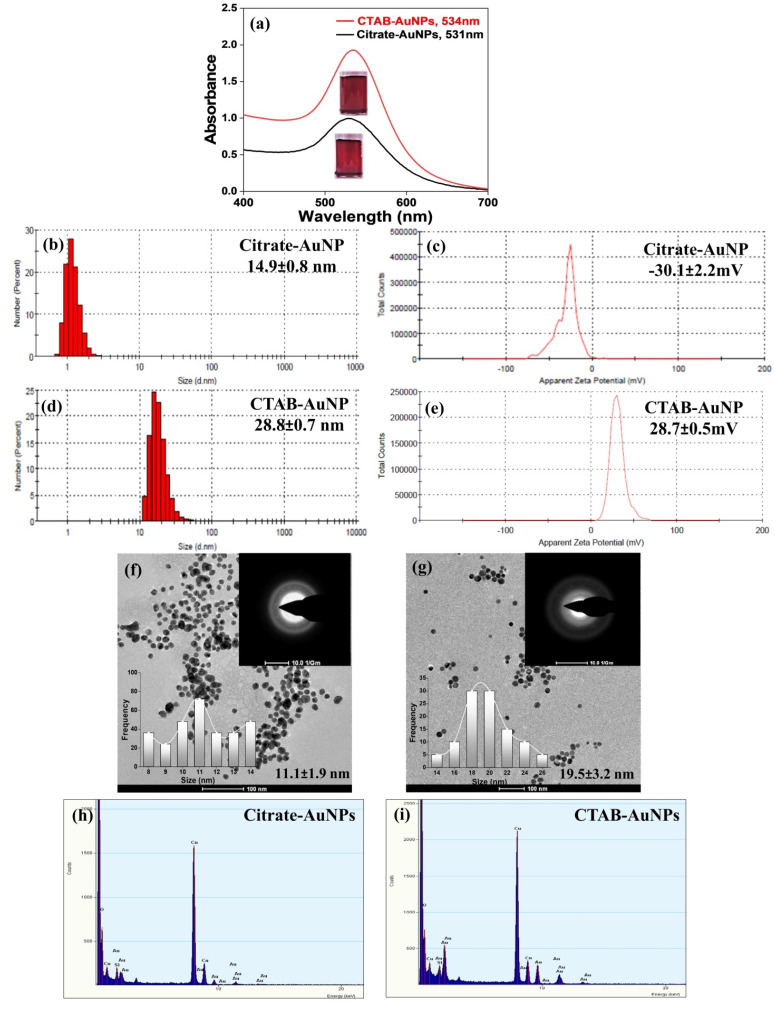
Characterization of the AuNPs. (**a**) UV–Vis absorption spectra of the synthesized citrate-AuNPs and CTAB-AuNPs (insets are the digital photographs of the corresponding AuNPs). (**b**,**d**) DLS and (**c**,**e**) zeta potentials of the citrate-AuNPs and CTAB-AuNPs, respectively. (**f**,**g**) The TEM micrographs (top-right insets are the SAED patterns, bottom-left insets are the TEM size distribution of the AuNPs) and (**h**,**i**) EDAX of the citrate-AuNPs and CTAB-AuNPs, respectively.

From the absorption studies, it was observed that the prepared citrate-AuNPs and CTAB-AuNPs showed characteristic surface plasmon resonance peaks at 531 and 534 nm respectively, which confirmed the successful synthesis of citrate-AuNPs and CTAB-AuNPs. From the DLS and zeta potential studies, the hydrodynamic size of citrate-AuNPs and CTAB-AuNPs was found to be ~14.9 ± 0.8 and 28.8 ± 0.7 nm, respectively. Zeta potential studies suggested a negative zeta potential of ~−30.1 ± 2.2 mV due to the presence of citrate on the AuNP surface. Furthermore, CTAB-AuNPs showed a positive zeta potential of ~28.7 ± 0.5 mV because of the capping of positively charged CTAB to the AuNP. Moreover, the polydispersity index (PDI) in both the AuNPs was found to be <0.3 (~0.25 for citrate-AuNPs and 0.21 for CTAB-AuNPs). All of these studies suggest the formation of stable colloidal solutions of citrate-AuNPs and CTAB-AuNPs (Table 2). TEM was conducted to evaluate the morphological characters and size of the synthesized AuNPs. The TEM micrographs showed the presence of uniformly distributed spherical AuNPs with sizes ranging from ~9–12 nm for citrate-AuNPs and ~17–23 nm for CTAB-AuNPs (Figure 1).

### 3.2. Citrate-AuNPs and CTAB-AuNPs Improves the Shoot and Root Proliferation and Biomass

**Figure 2:**
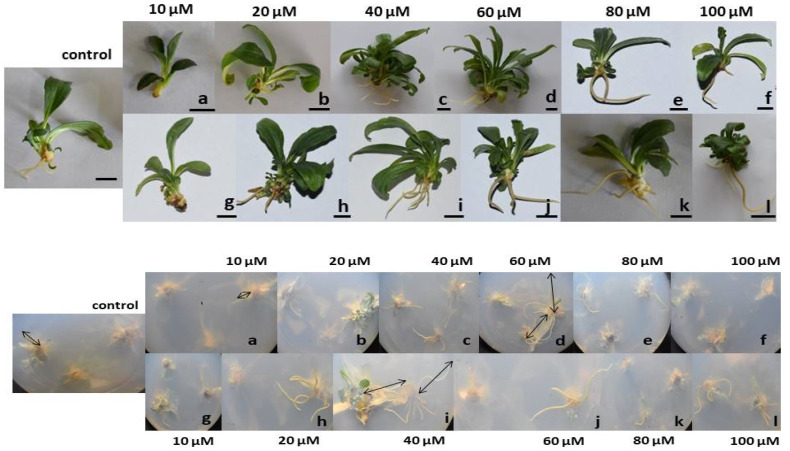
In vitro shoot and root proliferation in the citrate-AuNP and CTAB-AuNP treated *N. jatamansi* plants. **Upper panel**: shoot proliferation in the citrate-AuNP treated (**a**–**f**) and CTAB-AuNP treated (**g**–**l**) plants. Black lines show the root length. **Lower panel**: the root length and number of roots in the citrate-AuNP (**a**–**f**) and CTAB-AuNP treated (**g**–**l**) *N. jatamansi* plants. Scale bar = 1 cm.

**Figure 3:**
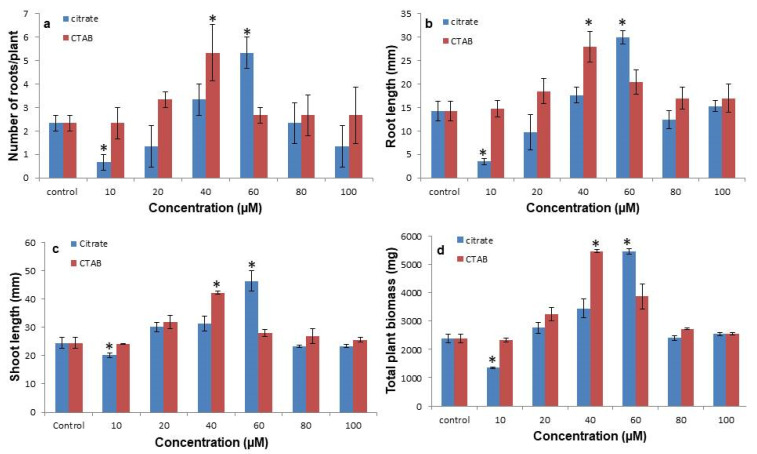
The effect of citrate-AuNPs and CTAB-AuNPs on the in vitro growth of *N. jatamansi*. (**a**) Effect on the number of roots per plant; (**b**) effect on root length (**c**) effect on shoot length; (**d**) effect on the total plant biomass. Error bar represents the standard error of three biological replicates. * Indicates significantly reduced and enhanced concentrations in comparison to the control.

Shoot and root proliferation was significantly affected by both the citrate-AuNPs and CTAB-AuNPs in a concentration dependent manner (Figure 2). After 30 days, it was observed that 10 µM concentrations of the citrate-AuNP treated in vitro plants had a significantly reduced root number (0.66 ± 0.33) and length (5.6 ± 1.1 mm) compared to the control plants, but there was no significant effect on the shoots. However, on increasing the concentration, the maximum root number was observed at 40 µM CTAB-AuNPs (5.33 ± 0.58), followed by 60 µM citrate-AuNPs (5.00 ± 0.67). However, the maximum root length (29.9 ± 1.5 mm) and shoot length (46.4 ± 3.7 mm) was recorded at 60 µM citrate-AuNPs followed by 40 µM CTAB-AuNPs (27.3 ± 4.8 mm and 42.2 ± 3.2 mm, respectively) and reduced thereafter at higher concentrations (Figure 3). In addition, both citrate-AuNPs and CTAB-AuNPs also significantly affected the total plant biomass, among which the minimum biomass was recorded in 10 µM citrate-AuNPs (1350 ± 31.4 mg), while the maximum biomass was recorded at the 60 µM citrate-AuNP (5460 ± 91.1 mg) and 40 µM concentration of the CTAB-AuNP (5480 ± 54.2 mg) treated plants.

### 3.3. Citrate-AuNPs and CTAB-AuNPs Treatment Improves Photosynthetic Pigment and Total Soluble Sugar Content

**Figure 4:**
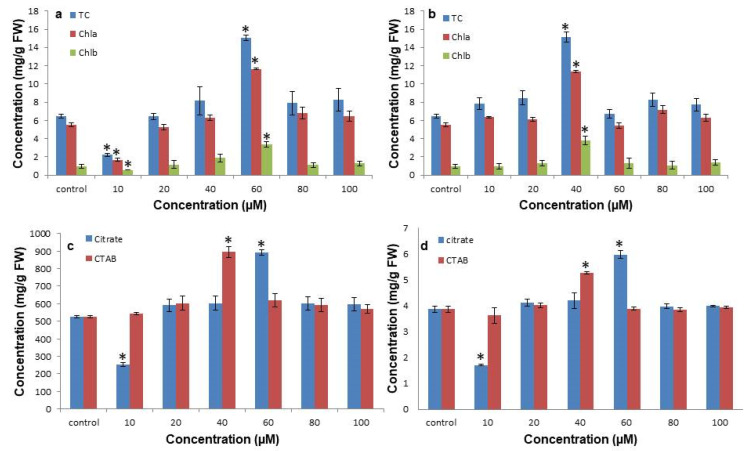
Effect of citrate-AuNPs and CTAB-AuNPs on in vitro photosynthetic pigment and total soluble sugar content in *N. jatamansi*. (**a**) The effect of citrate-AuNPs on the total chlorophyll, chlorophyll a and b content; (**b**) effect of CTAB-AuNPs on the total chlorophyll, chlorophyll a and b content; (**c**) effect of citrate-AuNPs and CTAB-AuNPs on the carotenoid content; (**d**) effect of citrate-AuNPs and CTAB-AuNPs on the total soluble sugar content. Error bar represents the standard error of three biological replicates. * Indicates significantly reduced and enhanced concentrations in comparison to the control.

Photosynthetic pigments were also affected differentially under the examined concentrations of the citrate-AuNPs and CTAB-AuNPs. Total chlorophyll, chlorophyll a and b content was significantly reduced (2.23 ± 0.17 mg/g, 1.66 ± 0.15 mg/g, 0.56 ± 0.02 mg/g fresh weight) at a 10 µM concentration of citrate-AuNPs, while at a 60 µM concentration, the pigments significantly increased (15.02 ± 0.29 mg/g, 11.66 ± 0.11 mg/g, 3.37 ± 0.29 mg/g fresh weight) in comparison to the control (6.46 ± 0.24 mg/g, 5.50 ± 0.21 mg/g, 0.96 ± 0.23 mg/g fresh weight) (Figure 4a). Furthermore, a 40 µM concentration of CTAB-AuNPs also increased the total chlorophyll and chlorophyll a and b content (15.11 ± 0.55 mg/g, 11.35 ± 0.15 mg/g, 3.76 ± 0.47 mg/g fresh weight) significantly compared to the control (Figure 4b). Similarly, the carotenoid contents were also differentially accumulated after the treatment of both citrate-AuNPs and CTAB-AuNPs (Figure 4c). The carotenoid content was significantly reduced (251.84 ± 10.23 mg/g fresh weight) at the 10 µM citrate-AuNP concentration compared to the control (525.55 ± 8.81 mg/g fresh weight). In contrast, a significantly higher content of carotenoids was observed at a 60 µM concentration of citrate-AuNPs (891.28 ± 14.73 mg/g fresh weight) and at a 40 µM concentration of CTAB-AuNPs (895.28 ± 30.57 mg/g fresh weight). The total soluble sugar content was also regulated in a concentration dependent manner of citrate-AuNPs and CTAB-AuNPs (Figure 4d). The minimum total soluble sugar content (1.71 ± 0.06 mg/g fresh weight) was observed at a 10 µM citrate-AuNP concentration, while the maximum content was observed at a 60 µM citrate-AuNP concentration (5.96 ± 0.14 mg/g fresh weight) followed by a 40 µM CTAB-AuNP concentration (5.26 ± 0.05 mg/g fresh weight) compared to the control (3.86 ± 0.1 mg/g fresh weight).

### 3.4. Citrate-AuNPs and CTAB-AuNPs Enhances Total Phenolics, Flavonoids, DPPH Radical Scavenging, and Superoxide Dismutase Enzymatic Activity

**Figure 5:**
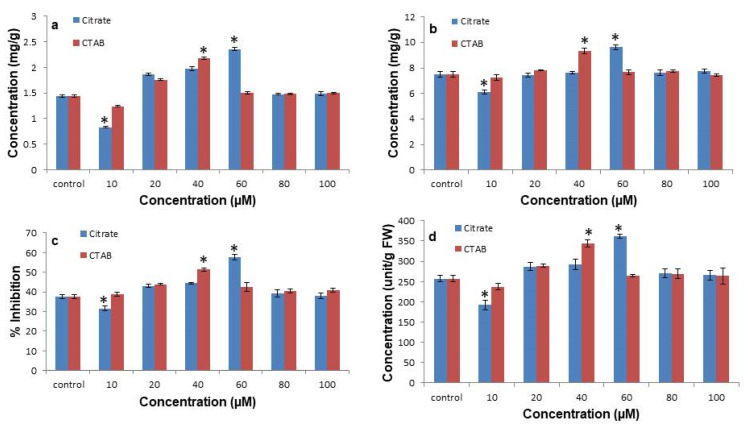
The impact of citrate-AuNPs and CTAB-AuNPs on the in vitro enhancement of antioxidant activity. (**a**) The effect of citrate-AuNPs and CTAB-AuNPs on the total phenolic content; (**b**) effect of citrate-AuNPs and CTAB-AuNPs on the total flavonoids; (**c**) effect of citrate-AuNPs and CTAB-AuNPs on the DPPH inhibition; (**d**) effect of citrate-AuNPs and CTAB-AuNPs on the SOD enzyme activity. Error bar represents the standard error of three biological replicates. * Indicates significantly reduced and enhanced concentrations in comparison to the control.

The treatment of citrate-AuNPs and CTAB-AuNPs was reported to increase the concentration of phenolic and flavonoid content in *N. jatamansi* (Figure 5a,b). A 10 µM concentration of citrate-AuNPs showed the lowest phenolic (0.83 ± 0.01 mg/g) and flavonoid (6.09 ± 0.22 mg/g) contents, while the highest content of phenolics (2.35 ± 0.02 mg/g) and flavonoids (9.62 ± 0.19 mg/g) was observed at a 60 µM concentration of citrate-AuNPs compared to the control (1.44 ± 0.02 mg/g, 7.51 ± 0.2 mg/g). Similarly, a significantly higher accumulation of phenolics (2.17 ± 0.02 mg/g) and flavonoids (9.33 ± 0.23 mg/g) was observed at a 40 µM CTAB-AuNPs concentration. DPPH radical scavenging activity was also improved after the treatment of both citrate-AuNPs and CTAB-AuNPs, in which the 10 µM and 60 µM concentrations of the citrate coated GNP treated plants showed the significantly minimum (31.5 ± 1.07%) and maximum (57.7 ± 1.4%) activity, respectively (Figure 5c). Similarly, at a 40 µM concentration of CTAB-AuNPs, the maximum radical scavenging activity (51.3 ± 0.75%) was observed compared to the control (37.67 ± 0.9%). Furthermore, the antioxidant enzyme superoxide dismutase activity was also improved with citrate-AuNP and CTAB-AuNP treatment, among which plants exposed to a 10 µM concentration of citrate-AuNPs had a significantly reduced (192.17 ± 11.5 unit/g) and those exposed to a 60 µM concentration of citrate-AuNP had a significantly enhanced (361.78 ± 5.11 unit/g) SOD activity (Figure 5d). In addition, enhanced SOD enzyme activity was observed at a 40 µM concentration of CTAB-AuNPs (344.44 ± 8.5 unit/g) compared to the control (257.1 ± 7.67 unit/g).

### 3.5. Citrate-AuNPs and CTAB-AuNPs Treatment Improves Endogenous Hormones Levels

**Figure 6:**
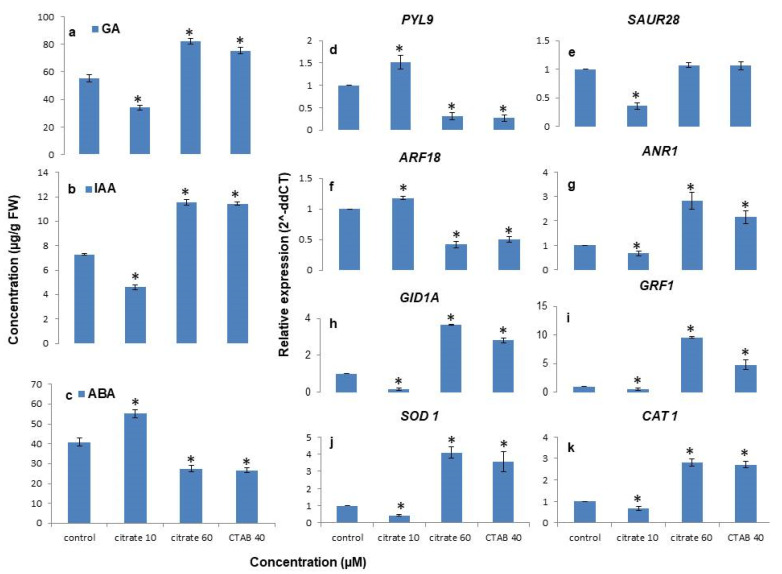
The effect of citrate-AuNPs and CTAB-AuNPs on the endogenous hormone level and relative transcript abundance of genes regulating antioxidant enzyme activities. (**a**) endogenous GA3 content; (**b**) endogenous IAA content; (**c**) endogenous ABA content; (**d**–**k**) relative transcript abundance of genes regulating endogenous hormone contents and antioxidant enzyme activities. Error bar represents the standard error of three biological replicates. * Indicates significant difference at *p* < 0.05 with respective control.

Citrate-AuNP and CTAB-AuNP treatment were also found to improve endogenous hormone levels under in vitro conditions in *N. jatamansi* (Figure 6). The IAA and GA3 levels were found to be significantly reduced in a 10 µM citrate-AuNP concentration (4.59 ± 0.23 µg/g, 33.94 ± 1.7 µg/g), whereas their levels improved significantly at a 60 µM concentration of citrate-AuNPs (11.55 ± 0.21 µg/g, 82.24 ± 2.1 µg/g) and a 40 µM concentration of CTAB-AuNPs (11.44 ± 0.13 µg/g, 75.4 ± 2.2 µg/g) compared to the control (7.27 ± 0.07 µg/g, 55.37 ± 2.5 µg/g). Furthermore, endogenous ABA content was significantly enhanced at a 10 µM concentration (55.22 ± 2.09 µg/g) of citrate-AuNPs. In contrast, endogenous ABA levels were significantly reduced under the 60 µM concentration of citrate-AuNPs (27.48 ± 1.52 µg/g) and the 40 µM concentration of (26.58 ± 1.18 µg/g) of the CTAB-AuNP treated plants compared to the control (40.84 ± 1.98 µg/g).

### 3.6. Citrate-AuNPs and CTAB-AuNPs Modulates Genes Regulating Hormone Content and Antioxidant Enzyme Activities

The relative expression of transcripts regulating the growth, hormonal profile, and antioxidant activity were differentially regulated by citrate-AuNPs and CTAB-AuNPs (Figure 6). It was observed that the expression of the ABA receptor, PYL9, increased 1.5-fold under the 10 µM citrate-AuNPs, whereas it was severely downregulated (3-fold) under the 60 µM citrate-AuNPs and the 40 µM CTAB-AuNPs in comparison to the control. However, small auxin upregulated RNA, *SAUR28* showed no variation in the transcript expression under both 60 µM citrate-AuNPs and 40 µM CTAB-AuNPs, but its expression was significantly declined under the 10 µM citrate-AuNP treatment. In contrast, the auxin response factor, ARF18 was significantly downregulated under both the 60 µM citrate-AuNP and 40 µM CTAB-AuNP concentrations, while significant upregulation was observed under the 10 µM concentrations of citrate-AuNPs. Furthermore, the MADS box transcription factor (*ANR1*), GA3 receptor (*GID1A*), growth regulating factor (*GRF1*), SOD, and CAT were significantly upregulated under both the 60 µM citrate-AuNP and 40 µM CTAB-AuNP concentration, whereas they were severely downregulated under the 10 µM concentration of citrate-AuNPs in comparison to the control. 

## 4. Discussion

In past few years, the application of nanoparticles has immensely increased in agriculture and plant tissue culture for the commercial production of various plants and their secondary metabolites [27,28]. The size and concentration of nanoparticles could play an imperative role in growth and development in a species-specific manner. A model was predicted to quantify engineered gold nanoparticles in water (0.14 μg/L) and soil (5.99 μg/kg) [29]. Other than a few studies, the overall toxic effect of gold nanoparticles was observed to be higher than 100 mg/L with particle sizes less than 5 nm [30], though the underlying mechanism is still not clearly understood. However, surface modifications could stabilize nanomaterials, though under natural conditions, the chemistry of the coating can change, affecting the properties and biocompatibility of nanoparticles [13]. The majority of studies on the toxic effect of nanoparticles have focused on animals and bacteria, and is significantly less available on plants [30]. Furthermore, the mechanism for the biological action of nanoparticles in plant development and antioxidant activity is yet to be determined. Therefore, it is very important to explore the effect of metallic as well as non-metallic nanoparticles on plants, especially medicinal plants. The size and concentration of nanoparticles could be imperative in the growth and development in a species-specific manner. Nanoparticles could enter the plasma membrane by creating some nano-scale holes of about 15–40 nm in diameter [31]. Although nanoparticles can aggregate in the growth medium, surface coatings are used and can play a key role in their stabilization. The physical interaction of nanoparticles with a plasma membrane can be influenced by positive or negative coatings. Logically, negatively charged nanoparticles should accumulate less on the cell surface due to electrostatic repulsion compared to the positively charged nanoparticles due to electrostatic attraction. However, negatively charged nanoparticles can overcome the hindrance by strong covalent bindings [32], and pass the cell membrane. The present study reflects that the smaller particle size, irrespective of the charge, may overcome electrostatic repulsion, and the metal ion gradient difference inside and outside the cell may play the key role in nanoparticle internalization.

In the present study, the application of citrate-AuNPs and CTAB-AuNPs significantly increased the shoot multiplication, root proliferation, total plant biomass, photosynthetic pigments, enzymatic, and non-enzymatic antioxidant levels and endogenous hormone levels in a dose dependent manner under in vitro conditions in *N. jatamansi*. It was observed earlier that green-synthesized biogenic Ca-AuNPs and PEGMA-AuNPs enhanced the fresh weight, dry weight, and total biomass in banana under in vitro conditions [33]. Furthermore, after 10 weeks of in vitro culture of *Lamprocapnos spectabilis*, treatment of AuNPs increased the shoot number, but reduced rooting [34]. Moreover, 10 mg/L of citrate-AuNPs were previously shown to influence all of the growth parameters of *Arabidopsis* [35]. In our findings, we observed that all the growth parameters as well as the physiological and biochemical activities were enhanced at an optimum concentration of 60 µM of citrate-AuNPs and 40 µM of CTAB-AuNPs, whereas negative regulation was observed at a 10 µM concentration of citrate-AuNPs. At a lower concentration, nanoparticles might also hinder the uptake of other nutrients and on increasing the concentration above the optimum due to a higher concentration of nanoparticles, no significant growth was observed. It was also reported that negatively charged gold nanoparticles might not be uptaken by the plant at lower doses [11]. Previous findings suggest that auxin works in an acid growth hypothesis, reducing the pH of the cell wall for cell elongation [36]; this might have helped the uptake of citrate-AuNPs. It was also confirmed by *SAUR28* gene expression, which promotes auxin-induced cell wall loosening through acidification by activating PM H^+^-ATPase and inhibiting PP2C.D family protein phosphatases, leading to cell expansion and enhanced solute uptake [36,37]. This gene expression somewhere also suggests that a lower concentration of citrate-AuNPs limits IAA and other nutrient uptake, leading to the retardation of growth and root development, which might be due to the accumulation of nanoparticles at the base of the plant. ARF family transcripts are known to regulate plant growth and stress tolerance; different sets of ARFs such as *ARF10*, *16*, *17*, and *18* are known for negatively regulation of root growth in plants, out of these, *ARF18* also has abiotic stress tolerance activity [38,39], thus we chose *ARF18* over the other ARFs. The decrease in the transcript levels of *ARF18* was also observed under the 60 µM citrate-AuNPs and 40 µM CTAB-AuNPs, which were similar to that in earlier studies [40,41]. Therefore, an increase in the shoot and root proliferation by the downregulation of *ARF18* is because it inhibits downstream auxins by forming homodimers, showing in response, a negative correlation with plant growth [42]. Moreover, the transcript levels of MADS–box transcription factor *ANR1* was found to be increased with the 60 µM citrate-AuNP and 40 µM CTAB-AuNP treatment. It was reported by previous researchers that *ANR1* acts as a key player in the root and shoot development by promoting auxin biosynthesis, transport, and accumulation, aside from influencing nitrate assimilation, amino acid metabolism, glycolysis, and TCA cycle [43,44]. A higher absorption of nitrate further improved the synthesis of phenolic compounds [45,46]. Citrate-coated gold nanoparticles positively influenced all of the growth parameters of Arabidopsis at a concentration of 10 mg/L [35].

Chlorophyll content is used to detect the photosynthetic performance of the plant, ultimately leading to overall plant growth and development. In addition, carotenoids prevent from photo-oxidation by absorbing excess light that is not absorbed by the chlorophyll pigments. Since chlorophyll and carotenoids are vital biomolecules for photosynthesis, understanding the alterations in their contents would provide a deep insight into plant adaptation to high altitude regions. In the current findings, the photosynthetic pigments (i.e., chlorophyll a, chlorophyll b, total chlorophyll, and carotenoids) showed a remarkable increase under the 60 µM citrate-AuNPs and 40 µM CTAB-AuNPs. This enhancement in the photosynthetic pigment content in the AuNP treated plants may be due to the upregulation of ROS-scavenging enzymes, which help detoxify ROS radicals [28]. The total soluble sugars including glucose, fructose, and sucrose are known to play key roles in plant metabolism, growth, and maintain ROS balance inside the cell [47]. In our study, the total soluble sugar content increased under the 60 µM citrate-AuNP and 40 µM CTAB-AuNP treated plants. This suggests that the nanoparticles induced cellular metabolism in a positive manner, which subsequently led to growth enhancement of the plant. An increase in the chlorophyll content and total soluble sugar was observed in in vitro grown banana under *Caralluma tuberculata* AuNPs (Ca-AuNPs) and polyethylene glycol methacrylate coated Ca-AuNP nanocomposites (PEGMA-AuNPs) [33]. 

Phenolics are important constituents of the plant cell, having redox properties to facilitate antioxidant activity, and they are the second largest group of secondary metabolites in plants. Similarly, flavonoids are secondary metabolites present inside the vacuole, and are responsible for plant development, photo protection, and antioxidant activity. Phenolic and flavonoid compounds prevent cells from oxidation by electrolyzing and purifying the ROS generated during the photosynthetic electron transport process [48]. In this work, the pattern of total phenolic content and flavonoid content increased in a concentration-dependent manner of citrate-AuNPs and CTAB-AuNPs. Phenolic biosynthesis primarily occurs through the arogenate/shikimate pathway generating phenylalanine, tyrosine, and tryptophan, which are precursors of auxin biosynthesis [46]. It was observed that nanoparticles stimulate phenolic compound synthesis, leading to an increase in systemic acquired resistance [49]. The DPPH radical scavenging activity is widely used to determine the antioxidant activity of the plant. Our results are in accordance with [33], where there was increased phenol, flavonoid, and DPPH radical activity under the Ca-AuNPs and PEGMA-AuNPs in the in vitro grown banana. Other researchers under in vitro conditions also observed similar results [19,50]. SOD provides a first line of defense molecule against the oxidative stress in cells, catalyzing the dismutation of superoxide radical (O_2_^•−^) into hydrogen peroxide (H_2_O_2_) [51]. SOD is the primary enzyme of ROS scavenging, which converts free radical oxygen to H_2_O_2_, and then CAT further converts this H_2_O_2_ into water and oxygen to mitigate the toxic effects. In this study, SOD activity was reported to be higher in the 60 µM citrate-AuNP and 40 µM CTAB-AuNP treatments. Enhanced SOD and peroxidase activities were observed earlier in the AuNP treated callus cultures of *Prunella vulgaris* [19]. The relative expression levels of genes regulating the SOD and CAT activity also showed a higher expression under the 60 µM citrate-AuNP and 40 µM CTAB-AuNP treatment. It was reported earlier that long-term exposure of different organs of rats with AuNPs increased the SOD and CAT activity, leading to a reduction in energy metabolism [52].

Endogenous hormones mainly play an acute role in the growth and stress adaptation of plants under critical conditions. Endogenous ABA is a growth retardant that leads to senescence and is also known to regulate stress adaptation in plants. A major form of endogenous auxin is IAA, and it is known to play a major role in primary root initiation. In our study, we assessed the endogenous IAA, GA3, and ABA levels to correlate the growth of gold nanoparticle treated *N. jatamansi*. The 60 µM citrate-AuNP and 40 µM CTAB-AuNP treatments enhanced the level of endogenous hormones GA3 and IAA, where there was increased plant growth. Similar to our results, *Musa* cultures supplemented with Ca-AuNPs and PEGMA-AuNPs showed increased IAA levels, in correlation with increased growth [33]. A positive correlation of endogenous IAA and GA with the rooting and plant growth of *Gerbera jamesonii* was also observed under the treatment of Se-NPs [53]. Furthermore, the expression of *GRF1* and *GID1A* was also found to increase under the 60 µM citrate-AuNP and 40 µM CTAB-AuNP treatments, which showed that AuNPs regulate the endogenous hormone levels in in vitro plant growth. It was previously shown that *GRF1* is a transcriptional activator that interacts with *GIF* (GRF INTERACTING FACTOR) and DELLA transcription factors to modulate cell proliferation, leading to leaf expansion [54,55]. Functional studies also revealed the key regulatory role of *GRF1* in plant growth and development, cell signaling, and phytohormone biosynthesis [56]. Similarly, it was observed that DELLA proteins interact with GA receptor GID1A proteins. Gibberellins induce stem elongation through binding to its receptor GID1A, which after binding to GA represses the expression of the GA inhibitor DELLA [57]. The DELLA domain mediates GA-dependent GAI interaction and GAI DELLA functions as the receiver for the activated GID1A receptor, thus promoting gibberellic acid signaling in plants [58]. The ROS showed positive regulation with ABA signaling, which plays a key role in the plant’s lateral root development in the exposure to environmental stress [59]. ABA was found to regulate root growth in a concentration dependent manner, where a higher concentration reduced the root growth and lower concentration induced the root growth [60]. A higher expression of *RCAR1/PYL9*, a cytoplasm and nuclear ABA receptor, was also found with the 60 µM citrate-AuNP and 40 µM CTAB-AuNP treatments in the present study. It was previously shown that *PYL9* directly interacted with *MYB77* and *MYB44*, and regulated auxin responsive genes to improve primary root growth and lateral root formation [61]. Further detailed studies on the mechanism of action of citrate-AuNPs and CTAB-AuNPs will provide a much clearer arena of the effect of AuNPs in plant growth and development.

## 5. Conclusions

From the present investigation, it can be concluded that applying 60 µM oof citrate-AuNPs and 40 µM of CTAB-AuNPs in the in vitro culture medium will enhance the shoot and root proliferation as well as the antioxidant properties of *N. jatamansi* in a concentration dependent manner. The outcomes of the photosynthetic pigments, antioxidant enzyme activities, phenolic compounds, internal hormone levels, and transcript analysis clearly indicated that the citrate-AuNPs and CTAB-AuNPs modulated the antioxidant mechanism and production of phenolic derivatives, thus restraining ROS production. Furthermore, the datasets obtained from the in vitro experimental findings clearly indicate that citrate-AuNPs and CTAB-AuNPs stimulate genes regulating endogenous hormone levels and plant growth. Thus, the current investigations revealed that charged gold nanoparticles can be further used in plant tissue culture medium for rapid and efficient micropropagation and for the mitigation of ROS production under high altitude conditions.

## Figures and Tables

**Table 1:** The list of primers used in the present study.

Primer Name	Sequence	Product Length (bp)
*PYL9_F*	CAGGAACGATGGTGATTGAATC	61
*PYL9_R*	TCCTCTTTAGTGTTTCCATCAGG	
*ARF18_F*	AGAGAAGGTTCACTGGCACT	150
*ARF18_R*	TGTAAGGTTCGATTTCCCATGG	
*SAUR28_F*	AGCTGTTTACGTTGGAGAGAA	150
*SAUR28_R*	CTGCAAGGAATTGTGAGACCA	
*ANR1_F*	CAGCCAGACAAGTGACATTTTC	100
*ANR1_R*	AAAATGATGAGAGCAACTTCAGC	
*GID1A_F*	GATCTCTATCGTCGCCTTCC	50
*GID1A_R*	TAGAGATTGGTATTGGCGAGCA	
*GRF1_F*	CACAGGCTTTCTTGAACGGT	178
*GRF1_R*	TCAACATGTGGGATGGAATTGT	
*SOD_F*	CAGCAGGATTGTAATGGGGTC	100
*SOD_R*	CTTTCTGGCCTTGCACCTG	
*CAT_F*	CTCCTCATCCCTGTGCATGA	60
*CAT_R*	TGTGCCCATCATAATAATCACCA	
*Actin_F*	TCAAATCACGACCGGCCATA	100
*Actin_R*	TTCCGGTGATGGAGTCACTCA	

**Table 2:** The DLS and zeta potentials of the citrate-AuNPs and CTAB-AuNPs.

S. No.	Sample	Hydrodynamic Size (nm)	Zeta Potential (mV)	PDI
1	**Citrate-AuNPs**	14.9 ± 0.8	−30.1 ± 2.2	0.25 ± 0.03
2	**CTAB-AuNPs**	28.8 ± 0.7	28.7 ± 0.5	0.21 ± 0.01

Values expressed as the mean ± SE of three technical replicates.

## Data Availability

The data are contained within the article and supplementary materials.

## Supplementary Materials

The following supporting information can be downloaded at: https://www.mdpi.com/article/10.3390/antiox11101962/s1, Figure S1: Contig sequences of *Nardostachys jatamansi* (24)*. PYL9*, *ARF18*, *ANR1*, *SAUR28*, *GRF1*, and *GID1A* were extracted from the SRR1241890 database. *CAT1* and *SOD1* were extracted from the SRR11184224 database.

## References

[1] Chauhan K.H., Oli S., Bisht K.A., Meredith C., Leaman D. (2021). Review of the biology, uses and conservation of the critically endangered endemic Himalayan species Nardostachys jatamansi (Caprifoliaceae). Biodivers. Conserv..

[2] Bose B., Kumaria S., Tandon P. (2022). Physiological insights into the role of temperature and light conditions on in vitro growth, membrane thermostability and antioxidative activity of Nardostachys jatamansi, an IUCN Red-listed critically endangered therapeutic plant. S. Afr. J. Bot..

[3] Dhiman N., Bhattacharya A. (2020). Nardostachys jatamansi (D. Don) DC.-Challenges and opportunities of harnessing the untapped medicinal plant from the Himalayas. J. Ethnopharmacol..

[4] Bose B., Tripathy D., Chatterjee A., Tandon P., Kumaria S. (2019). Secondary metabolite profiling, cytotoxicity, anti-inflammatory potential and in vitro inhibitory activities of Nardostachys jatamansi on key enzymes linked to hyperglycemia, hypertension and cognitive disorders. Phytomedicine.

[5] Dhiman N., Devi K., Bhattacharya A. (2021). Development of low cost micropropagation protocol for Nardostachys jatamansi: A critically endangered medicinal herb of Himalayas. S. Afr. J. Bot..

[6] Foyer C.H., Hanke G. (2022). ROS production and signalling in chloroplasts: Cornerstones and evolving concepts. Plant J..

[7] Mittler R., Zandalinas S.I., Fichman Y., Van Breusegem F. (2022). Reactive oxygen species signalling in plant stress responses. Nat. Rev. Mol. Cell Biol..

[8] Rajput V.D., Minkina T., Suskova S., Mandzhieva S., Tsitsuashvili V., Chapligin V., Fedorenko A. (2018). Effects of copper nanoparticles (CuO NPs) on crop plants: A mini review. BioNanoScience.

[9] Anik M.I., Mahmud N., Al Masud A., Hasan M. (2022). Gold nanoparticles (GNPs) in biomedical and clinical applications: A review. Nano Sel..

[10] Venzhik Y.V., Moshkov I.E., Dykman L.A. (2021). Gold Nanoparticles in Plant Physiology: Principal Effects and Prospects of Application. Russ. J. Plant Physl..

[11] Milewska-Hendel A., Gepfert W., Zubko M., Kurczyńska E. (2022). Morphological, Histological and Ultrastructural Changes in *Hordeum vulgare* (L.) Roots That Have Been Exposed to Negatively Charged Gold Nanoparticles. Appl. Sci..

[12] Mourdikoudis S., Pallares R.M., Thanh N.T. (2018). Characterization techniques for nanoparticles: Comparison and complementarity upon studying nanoparticle properties. Nanoscale.

[13] Wu H., Huang L., Rose A., Grassian V.H. (2020). Impact of surface adsorbed biologically and environmentally relevant coatings on TiO 2 nanoparticle reactivity. Environ. Sci. Nano.

[14] Dar A.I., Walia S., Acharya A. (2016). Citric acid-coated gold nanoparticles for visual colorimetric recognition of pesticide dimethoate. J. Nanopart. Res..

[15] Dar A.I., Abidi S.M., Randhawa S., Joshi R., Kumar R., Acharya A. (2022). Protein-Cloaked Nanoparticles for Enhanced Cellular Association and Controlled Pathophysiology via Immunosurveillance Escape. ACS Appl. Mater. Interfaces.

[16] Murashige T., Skoog F. (1962). A revised medium for rapid growth and bio assays with tobacco tissue cultures. Physiol. Plant..

[17] Arnon D.I. (1949). Copper enzymes in isolated chloroplasts. Polyphenoloxidase in Beta vulgaris. Plant Physiol..

[18] Joshi R., Ramanarao M.V., Baisakh N. (2013). Arabidopsis plants constitutively overexpressing a myo-inositol 1-phosphate synthase gene (SaINO1) from the halophyte smooth cordgrass exhibits enhanced level of tolerance to salt stress. Plant Physiol. Biochem..

[19] Fazal H., Abbasi B.H., Ahmad N., Ali M. (2016). Elicitation of medicinally important antioxidant secondary metabolites with silver and gold nanoparticles in callus cultures of *Prunella vulgaris* L.. Appl. Biochem. Biotechnol..

[20] Ahmed M., Ji M., Qin P., Gu Z., Liu Y., Sikandar A., Iqbal M.F., Javeed A. (2019). Phytochemical screening, total phenolic and flavonoids contents and antioxidant activities of *Citrullus colocynthis* L. and *Cannabis sativa* L.. Appl. Ecol. Environ. Res..

[21] Dhindsa R.S., Plumb-Dhindsa P., Thorpe T.A. (1981). Leaf senescence: Correlated with increased levels of membrane permeability and lipid peroxidation, and decreased levels of superoxide dismutase and catalase. J. Exp. Bot..

[22] Pan X., Welti R., Wang X. (2010). Quantitative analysis of major plant hormones in crude plant extracts by high-performance liquid chromatography–mass spectrometry. Nat. Protoc..

[23] Ghawana S., Paul A., Kumar H., Kumar A., Singh H., Bhardwaj P.K., Rani A., Singh R.S., Raizada J., Singh K. (2011). An RNA isolation system for plant tissues rich in secondary metabolites. BMC Res. Notes.

[24] Dhiman N., Kumar A., Kumar D., Bhattacharya A. (2020). De novo transcriptome analysis of the critically endangered alpine Himalayan herb Nardostachys jatamansi reveals the biosynthesis pathway genes of tissue-specific secondary metabolites. Sci. Rep..

[25] Gomez K.A., Gomez A.A. (1984). Statistical Procedures for Agricultural Research.

[26] Joshi R., Bhattacharya P., Sairam R.K., Sathee L., Chinnusamy V. (2020). Identification and characterization of NADH kinase-3 from a stress-tolerant wild mung bean species (*Vigna luteola* (Jacq.) Benth.) with a possible role in waterlogging tolerance. Plant Mol. Biol. Rep..

[27] Asl K.R., Hosseini B., Sharafi A., Palazon J. (2019). Influence of nano-zinc oxide on tropane alkaloid production, h6h gene transcription and antioxidant enzyme activity in *Hyoscyamus reticulatus* L. hairy roots. Eng. Life Sci..

[28] Singh A., Sengar R.S., Rajput V.D., Minkina T., Singh R.K. (2022). Zinc Oxide Nanoparticles Improve Salt Tolerance in Rice Seedlings by Improving Physiological and Biochemical Indices. Agriculture.

[29] Tiede K., Hassellöv M., Breitbarth E., Chaudhry Q., Boxall A.B. (2009). Considerations for environmental fate and ecotoxicity testing to support environmental risk assessments for engineered nanoparticles. J. Chromatogr..

[30] Siegel J., Záruba K., Švorčík V., Kroumanová K., Burketová L., Martinec J. (2018). Round-shape gold nanoparticles: Effect of particle size and concentration on Arabidopsis thaliana root growth. Nanoscale Res. Lett..

[31] Zhu Z.J., Wang H., Yan B., Zheng H., Jiang Y., Miranda O.R., Rotello V.M., Xing B., Vachet R.W. (2012). Effect of surface charge on the uptake and distribution of gold nanoparticles in four plant species. Envir. Sci. Technol..

[32] Sun H., Lei C., Xu J., Li R. (2021). Foliar uptake and leaf-to-root translocation of nanoplastics with different coating charge in maize plants. J. Hazard. Mater..

[33] Anwar N., Wahid J., Uddin J., Khan A., Shah M., Shah S.A., Subhan F., Khan M.A., Ali K., Rauf M. (2021). Phytosynthesis of poly (ethylene glycol) methacrylate-hybridized gold nanoparticles from C. tuberculata: Their structural characterization and potential for in vitro growth in banana. Vitr. Cell. Dev. Biol..

[34] Kulus D., Tymoszuk A., Jedrzejczyk I., Winiecki J. (2022). Gold nanoparticles and electromagnetic irradiation in tissue culture systems of bleeding heart: Biochemical, physiological, and (cyto) genetic effects. Plant Cell Tissue Organ Cult..

[35] Ferrari E., Barbero F., Busquets-Fité M., Franz-Wachtel M., Köhler H.R., Puntes V., Kemmerling B. (2021). Growth-Promoting Gold Nanoparticles Decrease Stress Responses in Arabidopsis Seedlings. Nanomaterials.

[36] Stortenbeker N., Bemer M. (2019). The SAUR gene family: The plant’s toolbox for adaptation of growth and development. J. Exp. Bot..

[37] Spartz A.K., Lor V.S., Ren H., Olszewski N.E., Miller N.D., Wu G., Spalding E.P., Gray W.M. (2017). Constitutive expression of Arabidopsis SMALL AUXIN UP RNA19 (SAUR19) in tomato confers auxin-independent hypocotyl elongation. Plant Physiol..

[38] Tang Y., Du G., Xiang J., Hu C., Li X., Wang W., Sui J. (2022). Genome-wide identification of auxin response factor (ARF) gene family and the miR160-ARF18-mediated response to salt stress in peanut (*Arachis hypogaea* L.). Genomics.

[39] Kou X., Zhao X., Wu B., Wang C., Wu C., Yang S., Xue Z. (2022). Auxin response factors are ubiquitous in plant growth and development, and involved in crosstalk between plant hormones: A review. Appl. Sci..

[40] Wójcikowska B., Gaj M.D. (2017). Expression profiling of AUXIN RESPONSE FACTOR genes during somatic embryogenesis induction in Arabidopsis. Plant Cell Rep..

[41] Quintana-Escobar A.O., Nic-Can G.I., Avalos R.M.G., Loyola-Vargas V.M., Gongora-Castillo E. (2019). Transcriptome analysis of the induction of somatic embryogenesis in Coffea canephora and the participation of ARF and Aux/IAA genes. Peer J..

[42] Liu J., Hua W., Hu Z., Yang H., Zhang L., Li R., Deng L., Sun X., Wang X., Wang H. (2015). Natural variation in ARF18 gene simultaneously affects seed weight and silique length in polyploid rapeseed. Proc. Natl. Acad. Sci. USA.

[43] Sun C.H., Yu J.Q., Wen L.Z., Guo Y.H., Sun X., Hao Y.J., Hu D.G., Zheng C.S. (2018). Chrysanthemum MADS-box transcription factor CmANR1 modulates lateral root development via homo-/heterodimerization to influence auxin accumulation in Arabidopsis. Plant Sci..

[44] Sun C.H., Wang J.H., Gu K.D., Zhang P., Zhang X.Y., Zheng C.S., Hu D.G., Ma F. (2021). New insights into the role of MADS-box transcription factor gene CmANR1 on root and shoot development in chrysanthemum (*Chrysanthemum morifolium*). BMC Plant Biol..

[45] Rivero-Montejo S.D.J., Vargas-Hernandez M., Torres-Pacheco I. (2021). Nanoparticles as novel elicitors to improve bioactive compounds in plants. Agriculture.

[46] Shoja A.A., Çirak C., Ganjeali A., Cheniany M. (2022). Stimulation of phenolic compounds accumulation and antioxidant activity in in vitro culture of Salvia tebesana Bunge in response to nano-TiO2 and methyl jasmonate elicitors. Plant Cll Tissue Organ Cult..

[47] Couée I., Sulmon C., Gouesbet G., El Amrani A. (2006). Involvement of soluble sugars in reactive oxygen species balance and responses to oxidative stress in plants. J. Exp. Bot..

[48] Shen Y., Jin L., Xiao P., Lu Y., Bao J. (2009). Total phenolics, flavonoids, antioxidant capacity in rice grain and their relations to grain color, size and weight. J. Cereal Sci..

[49] Singh A., Dwivedi P. (2018). Methyl-jasmonate and salicylic acid as potent elicitors for secondary metabolite production in medicinal plants: A review. J. Pharmacogn. Phytochem..

[50] Kokina I., Gerbreders V., Sledevskis E., Bulanovs A. (2013). Penetration of nanoparticles in flax (*Linum usitatissimum* L.) calli and regenerants. J. Biotechnol..

[51] Ighodaro O.M., Akinloye O.A. (2018). First line defence antioxidants-superoxide dismutase (SOD), catalase (CAT) and glutathione peroxidase (GPX): Their fundamental role in the entire antioxidant defence grid. Alexandria Med. J..

[52] Ferreira G.K., Cardoso E., Vuolo F.S., Michels M., Zanoni E.T., Carvalho-Silva M., Gomes L.M., Dal-Pizzol F., Rezin G.T., Streck E.L. (2015). Gold nanoparticles alter parameters of oxidative stress and energy metabolism in organs of adult rats. Biochem. Cell Biol..

[53] Khai H.D., Mai N.T.N., Tung H.T., Luan V.Q., Cuong D.M., Ngan H.T.M., Chau N.H., Buu N.Q., Vinh N.Q., Dung D.M. (2022). Selenium nanoparticles as in vitro rooting agent, regulates stomata closure and antioxidant activity of gerbera to tolerate acclimatization stress. Plant Cell Tissue Organ Cult..

[54] Lu Y., Meng Y., Zeng J., Luo Y., Feng Z., Bian L., Gao S. (2020). Coordination between GROWTH-REGULATING FACTOR1 and GRF-INTERACTING FACTOR1 plays a key role in regulating leaf growth in rice. BMC Plant Biol..

[55] Lantzouni O., Alkofer A., Falter-Braun P., Schwechheimer C. (2020). GROWTH-REGULATING FACTORS interact with DELLAs and regulate growth in cold stress. Plant Cell.

[56] Piya S., Liu J., Burch-Smith T., Baum T.J., Hewezi T. (2020). A role for Arabidopsis growth-regulating factors 1 and 3 in growth–stress antagonism. J. Exp. Bot..

[57] Hauvermale A.L., Ariizumi T., Steber C.M. (2014). The roles of the GA receptors GID1a, GID1b, and GID1c in sly1-independent GA signaling. Plant Signal. Behav..

[58] Willige B.C., Ghosh S., Nill C., Zourelidou M., Dohmann E.M., Maier A., Schwechheimer C. (2007). The DELLA domain of GA INSENSITIVE mediates the interaction with the GA INSENSITIVE DWARF1A gibberellin receptor of Arabidopsis. The Plant Cell.

[59] Syu Y.Y., Hung J.H., Chen J.C., Chuang H.W. (2014). Impacts of size and shape of silver nanoparticles on Arabidopsis plant growth and gene expression. Plant Physiol. Biochem..

[60] Yang J., Cao W., Rui Y. (2017). Interactions between nanoparticles and plants: Phytotoxicity and defense mechanisms. J. Plant Interact..

[61] Xing L., Zhao Y., Gao J., Xiang C., Zhu J.K. (2016). The ABA receptor PYL9 together with PYL8 plays an important role in regulating lateral root growth. Sci. Rep..

