# Supplementary material for: Charged Gold Nanoparticles Promote In Vitro Proliferation in Nardostachys jatamansi by Differentially Regulating Chlorophyll Content, Hormone Concentration, and Antioxidant Activity
Source: Antioxidants (Basel). 2022 Sep 30;11(10):1962. doi: 10.3390/antiox11101962 (PMC9598260; doi:10.3390/antiox11101962)
Supplement: Supplementary file 1 [file antioxidants-11-01962-s001.zip › antioxidants-1922965-SI.pdf]

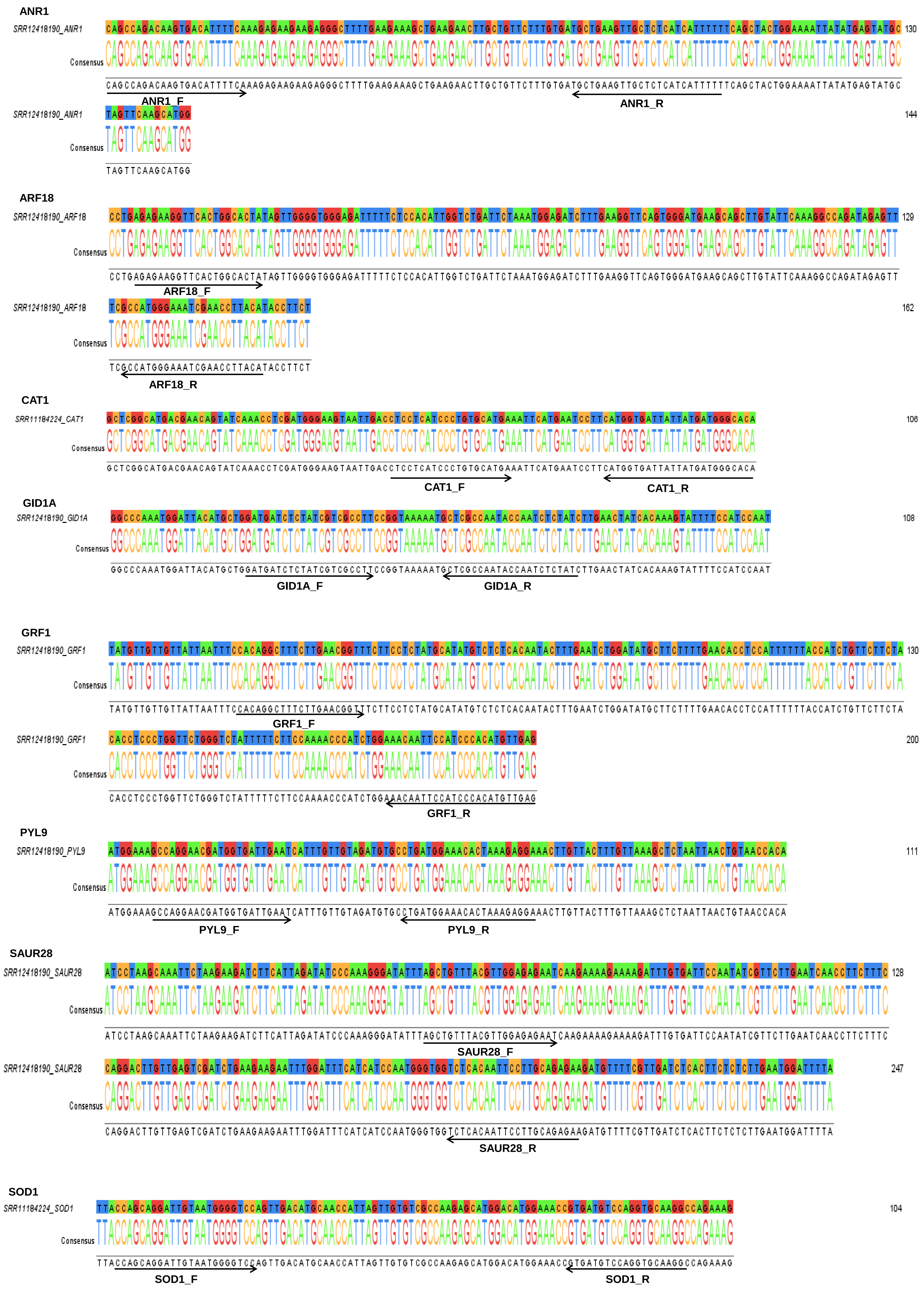

Supplementary figure S1. Contig sequences of *Nardostachys jatamansi* (Dhiman et al., 2020). *PYL9, ARF18, ANR1, SAUR28, GRF1* and *GID1A* were extracted from SRR1241890 database. *CAT1* and *SOD1* were extracted from SRR11184224 database.
